# Blood Flow Restriction Training Reduces Blood Pressure During Exercise Without Affecting Metaboreflex Activity

**DOI:** 10.3389/fphys.2018.01736

**Published:** 2018-12-04

**Authors:** Antonio Crisafulli, Rafael Riera de Farias, Paulo Farinatti, Karynne Grutter Lopes, Raffaele Milia, Gianmarco Sainas, Virginia Pinna, Girolamo Palazzolo, Azzurra Doneddu, Sara Magnani, Gabriele Mulliri, Silvana Roberto, Ricardo Brandão Oliveira

**Affiliations:** ^1^Sports Physiology Laboratory, The Department of Medical Sciences and Public Health, and International PhD in Innovation Sciences and Technologies, University of Cagliari, Cagliari, Italy; ^2^Laboratory of Physical Activity and Health Promotion, University of Rio de Janeiro, Rio de Janeiro, Brazil; ^3^Graduate Program in Exercise and Sport Sciences, University of Rio de Janeiro State, Rio de Janeiro, Brazil; ^4^Graduate Program in Physical Activity Sciences, Salgado de Oliveira University, Niterói, Brazil; ^5^Graduate Program in Clinical and Experimental Physiopathology, University of Rio de Janeiro State, Rio de Janeiro, Brazil; ^6^Laboratory of Vascular Biology, University of Rio de Janeiro State, Rio de Janeiro, Brazil; ^7^Laboratory of Active Living (LaVA), University of Rio de Janeiro State, Rio de Janeiro, Brazil

**Keywords:** blood flow restriction, ischemia, metaboreflex, exercise training, exercise pressor reflex, blood pressure

## Abstract

**Objective:** Blood flow restriction training (BFRT) has been proposed to induce muscle hypertrophy, but its safety remains controversial as it may increase mean arterial pressure (MAP) due to muscle metaboreflex activation. However, BFR training also causes metabolite accumulation that may desensitize type III and IV nerve endings, which trigger muscle metaboreflex. Then, we hypothesized that a period of BFR training would result in blunted hemodynamic activation during muscle metaboreflex.

**Methods:** 17 young healthy males aged 18–25 yrs enrolled in this study. Hemodynamic responses during muscle metaboreflex were assessed by means of postexercise muscle ischemia (PEMI) at baseline (T0) and after 1 month (T1) of dynamic BFRT. BFRT consisted of 3-min rhythmic handgrip exercise applied 3 days/week (30 contractions per minute at 30% of maximum voluntary contraction) in the dominant arm. On the first week, the occlusion was set at 75% of resting systolic blood pressure (always obtained after 3 min of resting) and increased 25% every week, until reaching 150% of resting systolic pressure at week four. Hemodynamic measurements were assessed by means of impedance cardiography.

**Results:** BFRT reduced MAP during handgrip exercise (T1: 96.3 ± 8.3 mmHg vs. T0: 102.0 ± 9.53 mmHg, *p* = 0.012). However, no significant time effect was detected for MAP during the metaboreflex activation (*P* > 0.05). Additionally, none of the observed hemodynamic outcomes, including systemic vascular resistance (SVR), showed significant difference between T0 and T1 during the metaboreflex activation (*P* > 0.05).

**Conclusion:** BFRT reduced blood pressure during handgrip exercise, thereby suggesting a potential hypotensive effect of this modality of training. However, MAP reduction during handgrip seemed not to be provoked by lowered metaboreflex activity.

## Introduction

Resistance training with low to moderate loads performed under blood flow restriction (BFR) has been shown to elicit muscle hypertrophy and strength gains (Kaijser et al., 1990; Abe et al., 2006; Manini and Clark, 2009; Takada et al., 2012). These effects have been associated to increased metabolite accumulation in the active skeletal muscle (Suga et al., 2012; Pearson and Hussain, 2015). On the other hand, the metabolite accumulation due to BFR also increases afferent signaling of group IV afferent muscle nerves (Nobrega et al., 2014). Together with central command and arterial/cardiopulmonary baroreceptors, groups III and IV skeletal muscle nerve afferents play an important role in mediating hemodynamic responses to exercise.

In addition, cardiovascular reflexes from group IV skeletal muscle afferents appear to be dysregulated in several cardio-metabolic diseases, such as obesity, metabolic syndrome, type 2 diabetes mellitus, chronic heart failure, or hypertension (Crisafulli et al., 2007, 2013; Piepoli et al., 2008; Sausen et al., 2009; Delaney et al., 2010; Roberto et al., 2012). The over-activation of signals originating from muscle type IV nerve endings has been suggested to be one of the beneficial effects of regular exercise upon chronic heart failure (Crisafulli et al., 2007, 2013; Piepoli et al., 2008). In fact, exercise training has been reported to reduce the metaboreflex activity (Wang et al., 2010).

Different approaches have been used to assess muscle metaboreflex activity (Alam and Smirk, 1937, 1938; Rowell et al., 1986; Crisafulli, 2017). In general, they involve BFR to the exercising muscles during or postexercise, thereby causing a mismatch in the oxygen supply-to-demand ratio. Consequently, an increased accumulation of metabolites occurs with the activation of group IV muscle afferents. Under physiological conditions, postexercise muscular ischemia (PEMI) activates afferent signals of group IV afferents in isolation from central command and muscle mechanoreflex (Crisafulli et al., 2011).

We could not find prior studies investigating the effects of BFR training on the hemodynamic responses induced by the metaboreflex. However, a recent study suggested that the exercise pressor reflex would be lowered after BFR training, therefore reducing the heart rate (HR) and blood pressure during the metaboreflex (Sundblad et al., 2018). Additionally, some other trials reported that BFR might have an acute hypotensive effect (Neto et al., 2015). However, potential mechanisms of hypotension after BFR training have not been investigated. In short, there is a lack of research explaining the cardiovascular effects of BFR training.

Considering the exercise-related reduction in the metaboreflex activity, as well as relatively recent findings in regard to blood pressure reduction after BFR, it is possible to speculate that a period of BFR training would result in attenuated blood pressure during the muscle metaboreflex activation. These data would have the following practical applications: (a) to help detecting hemodynamic improvements due to BFR training; (b) to demonstrate the safety of chronic BFR training and describe its hemodynamic effects.

Thus, the present study investigated whether BFR training would be capable to reduce the blood pressure and improve the vascular response during muscle metaboreflex activation in healthy subjects. We tested the hypothesis that a possible blood pressure reduction within metaboreflex activation would be due to lowered systemic vascular resistance (SVR). Additionally, we tested the hypothesis that BFR training might reduce the vasoconstriction mediated by the metaboreflex.

## Materials and Methods

### Sample

Sample size calculation was performed using the G-power software (Faul et al., 2007). A sample of 16 individuals was determined for an error probability of 0.05, effect size of 0.8, and power of 0.90 (1 – β). Initially, 39 volunteers enrolled in the study. Of these, 15 were excluded for not completing at least 75% of the planned training sessions. Another seven subjects were excluded due to low quality in their cardio-impedance tracing signals. Therefore, data of 17 male volunteers aged 18–25 years (21 ± 2 years; 1.73 ± 0.06 m; 76.3 ± 11.6 kg) were retained in the final analysis. All subjects were normotensive [117 ± 10 mmHg and 82 ± 9 mmHg (resting mean ± SD systolic and diastolic blood pressures, respectively)] and regularly practicing physical activity. None of the volunteers had history of cardiorespiratory or metabolic diseases or were under medications or supplements that might affect autonomic and hemodynamics responses. All participants signed informed consents and the experiment gained approval from the ethics board committee of the University of Rio de Janeiro State (process 69072916.8.0000.5282).

### Experimental Design

All subjects had hemodynamic responses assessed at rest, during handgrip exercise and during PEMI (metaboreflex activation), before (T0) and after 4 weeks (T1) of low intensity BFR training. All assessments took place within 1–3 days before T0 and after T1.

**FIGURE 1:**
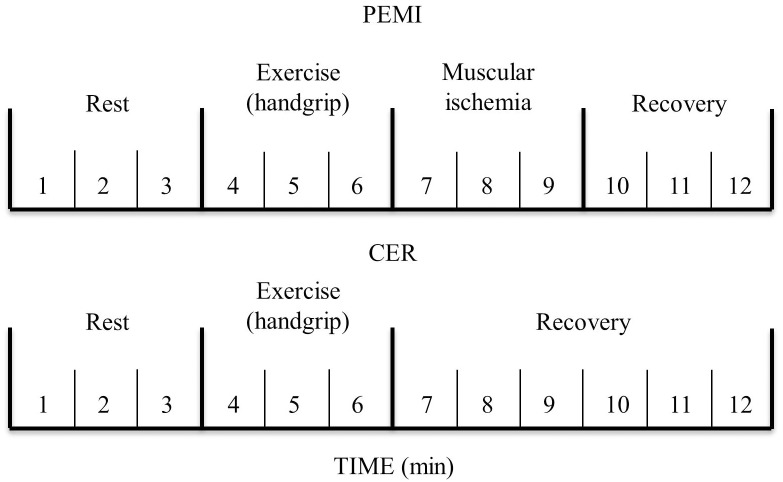
Metaboreflex assessment.

After a general medical examination, subjects remained 10 min at rest in a sit position before PEMI and control exercise recovery (CER) protocols. PEMI and CER were performed in a counterbalanced random order, with 10 min of resting between them. In the PEMI protocol, after 3 min of resting, the individuals performed rhythmic dynamic handgrip for another 3 min (30 contractions per min) with load corresponding to 30% of maximum voluntary contraction. Subsequently, 3 min of PEMI on the exercised arm was applied, by means of rapidly inflation (<3 s) of a tourniquet placed at the upper arm up to 50 mmHg above peak exercise systolic pressure. The cuff was inflated just at the cessation of exercise and was kept inflated for 3 min. After deflating the cuff, the individuals underwent additional 3 min recovery (total of 6 min). A manual sphygmomanometer (Welch^TM^ AllymTM DS44, Skaneateles Falls, NY, United States) was used to asses systolic and diastolic blood pressures (SBP and DPB) at every minute during, always in the non-dominant arm. This protocol has been shown to trap muscle metabolites in the exercising limb and to maintain stimulation of the metaboreceptors (Crisafulli et al., 2003, 2006, 2007, 2008). Moreover, this procedure allows isolating the metaboreflex activity from the activity due to central command and mechanoreflex activation, since during PEMI these two cardiovascular reflexes are not operating (Bastos et al., 2000; Crisafulli et al., 2011). In the CER protocol, the same rest-exercise protocol used for PEMI was performed, followed by 6-min recovery without tourniquet inflation (Figure 1 – Metaboreflex assessment). All experiments took place in the morning, in a temperature- controlled room (22°C, relative humidity 50%).

### Blood Flow Restriction (BFR) Training

Prior to the experimental conditions, maximal handgrip values were individually obtained through 5 maximum attempts with duration of 5 s interspersed with 1 min intervals. All measurements were recorded by using a previously calibrated MP150 Data Acquisition Systems module and the AcqKnowledge software (BIOPAC^TM^ Systems, Inc., Goleta, CA, United States). After all attempts, the maximal value was used to calculate the handgrip training load. One to 3 days after PEMI and CER assessments at baseline, the individuals began the supervised BFR training, 3 days per week during 4 weeks (therefore completing 12 sessions). As aforementioned, after 3 min seated at rest, 3 min of rhythmic handgrip exercise (30 contractions per min at 30% of maximum voluntary contraction) were performed with BFR being applied at the exercised arm. A visual inspection was allowed to the subjects in order to control their handgrip strength by means of the AcqKnowledge software. On the first week, the occlusion was set at 75% of resting SBP (always obtained after 3 min of resting) and increased 25% every week, until reaching 150% of resting SBP at week four.

### Hemodynamic Assessment

Hemodynamic assessments were performed by means of impedance cardiography (New Core^TM^, 2C Technologies Inc., Cagliari, Italy). The impedance method has been previously used in similar experimental settings (Crisafulli et al., 2003, 2007, 2011), and data acquisition procedures were described in detail in previous works by our group (Crisafulli et al., 2003, 2008). In short, the New Core device recorded impedance (Z_0_) and ECG traces on a secure digital memory card. The recorded Z_0_ and ECG traces were analyzed offline employing a digital chart recorder (ADInstruments^TM^, PowerLab 8sp, Castle Hill, Australia). The Z_0_ first derivative (dZ/dt) was calculated and the Sramek–Bernstein equation (Crisafulli et al., 2006) was employed to calculate beat-to-beat stroke volume (SV) values, as follows: SV = (VEPT•Z_0_^−1^)• dZ/dt_max_•VET; where VEPT is the volume of electrical participating tissue (determined using a nomogram based on sex, height, and body mass); Z_0_ is the thorax impedance at the end of cardiac diastole; dZ/dt_max_ is the maximal Z_0_ first derivative during cardiac systole; and VET is the left ventricular ejection time, calculated as the interval between the beginning and minimum deflection of dZ/dt trace during systole.

The pre-ejection period (PEP) was assessed as the time interval between the onset of electrocardiogram Q wave and the beginning of the widest deflection occurring in the dZ/dt trace (Crisafulli et al., 2000, 2003). The HR was calculated as the reciprocal of the electrocardiogram R–R interval and the cardiac output (CO) was obtained by multiplying SV and HR. Furthermore, diastolic time (DT) was measured by subtracting the sum of PEP and VET from the total cardiac cycle period. The mean systolic ejection rate (VER), which is an index of myocardial performance, was obtained by calculating the SV/VET ratio (Gledhill et al., 1994; Sanna et al., 2017). The ventricular filling rate (VFR), which is a measure of the mean rate of diastolic blood flux, was calculated by dividing SV by DT (Sanna et al., 2017).

The individuals were also connected to a standard manual sphygmomanometer (Welch Allym^TM^ DS44, Skaneateles Falls, NY, United States) to assess SBP and DBP, always in the non-dominant arm by the same trained researcher. The mean arterial blood pressure (MAP) was calculated using the formula proposed by Moran and co-workers (Moran et al., 1995). The systemic vascular resistance (SVR) was obtained by multiplying the MAP/CO ratio by 80, where 80 is a conversion factor to change units to standard resistance units. Blood pressure measurements were taken every minute by a single and experienced researcher.

### Data Analysis

The Shapiro-Wilk test revealed that all measured variables were normally distributed. Data are presented as mean ± SD. Hemodynamic data during PEMI and CER tests were averaged over 1 min. Values at the third minute of rest, at the third minute of exercise, and at the third minute of recovery in both protocols were considered for statistical analysis. In order to assess the metaboreflex activity, differences of all variables between PEMI and CER at the third minute of recovery were calculated (Crisafulli et al., 2013). Differences of outcomes between experimental situations were tested by 2-way ANOVA with repeated measures (factors: time and condition) followed by Bonferroni *post hoc* tests in the event of significant *F* ratios, while differences in deltas (Δ) for each main variable responses between T0 and T1 were assessed by *t*-tests for paired data. All calculations were performed using a commercially available software (GraphPad^TM^, Prism, La Jolla, CA, United States), and in all cases statistical significance was set at *P* ≤ 0.05.

## Results

Table 1 depicts resting hemodynamic outcomes before PEMI and CER, at T0 and T1. There was no significant difference across conditions and between T0 and T1 for HR, CO, MAP, SVR, VER, or VFR. A significant time effect (T0 vs. T1) was observed only for SV, which lowered after BFR training.

**Table 1:** Resting hemodynamic data before (T0) and after (T1) blood flow restriction in PEMI and CER (mean ± SD) (*n* = 17).

	T0	T1	*p*-value time effect	*p*-value condition effect	*p*-value interaction
HR (bpm)	PEMI 66.6 ± 9.2 CER 68.1 ± 11.5	PEMI 71.5 ± 11.7 CER 70.3 ± 10.8	0.181	0.954	0.609
SV (ml)	PEMI 135.5 ± 26.1 CER 122.3 ± 32.8	PEMI 115.5 ± 27.3^∗^ CER 112.8 ± 22.3^∗^	0.028	0.230	0.441
CO (L ⋅ min^−1^)	PEMI 9.01 ± 2.05 CER 8.36 ± 2.76	PEMI 8.28 ± 2.17 CER 7.87 ± 1.62	0.254	0.321	0.821
MAP (mmHg)	PEMI 96.4 ± 10.9 CER 95.0 ± 9.7	PEMI 91.5 ± 7.4 CER 92.8 ± 9.7	0.128	0.982	0.560
SVR (dynes ⋅ s^−1^ ⋅ cm^−5^)	PEMI 892.3 ± 202.7 CER 1004.8 ± 348.1	PEMI 957.9 ± 282.7 CER 977.8 ± 198.4	0.765	0.307	0.474
VER (ml ⋅ s^−1^)	PEMI 399.8 ± 73.1 CER 397.9 ± 89.3	PEMI 374.4 ± 85.0 CER 376.3 ± 86.1	0.253	0.990	0.931
VFR (ml ⋅ s^−1^)	PEMI 374.0 ± 249.0 CER 330.7 ± 183.2	PEMI 330.4 ± 188.3 CER 331.1 ± 188.9	0.664	0.668	0.658

*HR, heart rate; SV, stroke volume; CO, cardiac output; MAP, mean arterial pressure; SVR, systemic vascular resistance; VER, mean systolic ejection rate; VFR, ventricular filling rate. ^∗^Significantly different vs. T0.*

Table 2 presents data of main hemodynamic variables at the third minute of exercise in PEMI and CER, also at T0 and T1. Significant differences were not detected for HR, CO, SV, SVR, VFR, and VER between experimental conditions and between T0 and T1. On the other hand, a significant reduction in MAP was observed at T1 *vs.* T0 in both PEMI (101.7 ± 11.4 vs. 96.3 ± 8.3 mmHg) and CER (102.0 ± 9.5 vs. 95.7 ± 8.1 mmHg, *P* = 0.012 for time factor).

**Table 2:** Hemodynamic data values during the third minute of exercise (dynamic handgrip) of PEMI and CER tests before (T0) and after blood flow restriction protocol (T1).

	T0	T1	*p*-value time effect	*p*-value condition effect	*p*-value interaction
HR (bpm)	PEMI 69.6 ± 10.5 CER 71.3 ± 12.5	PEMI 73.4 ± 12.0 CER 75.0 ± 12.8	0.201	0.572	0.986
SV (ml)	PEMI 118.9 ± 27.9 CER 117.7 ± 32.5	PEMI 116.8 ± 29.3 CER 112.8 ± 24.9	0.617	0.710	0.841
CO (L ⋅ min^−1^)	PEMI 8.23 ± 2.00 CER 8.45. ± 2.86	PEMI 8.41 ± 1.88 CER 8.33 ± 1.73	0.954	0.894	0.775
MAP (mmHg)	PEMI 102.0 ± 9.53 CER 101.7 ± 11.4	PEMI 96.3 ± 8.3^∗^ CER 95.7 ± 8.1^∗^	0.012	0.947	0.844
SVR (dynes ⋅ s^−1^ ⋅ cm^−5^)	PEMI 1073.8 ± 378.2 CER 1041.4 ± 279.9	PEMI 962.6 ± 238.6 CER 953.3 ± 210.1	0.152	0.867	0.763
VER (ml ⋅ s^−1^)	PEMI 369.1 ± 89.3 CER 387.2 ± 98.8	PEMI 381.1 ± 93.1 CER 378.9 ± 86.8	0.934	0.723	0.651
VFR (ml ⋅ s^−1^)	PEMI 351.3 ± 170.2 CER 341.4 ± 193.9	PEMI 341.5 ± 121.2 CER 365.2 ± 193.6	0.867	0.869	0.689

*Values are mean ± SD. N = 17. HR, heart rate; SV, stroke volume; CO, cardiac output; MAP, mean arterial pressure; SVR, systemic vascular resistance; VER, mean systolic ejection rate; VFR, ventricular filling rate. ^∗^Significantly different vs. T0.*

**FIGURE 2:**
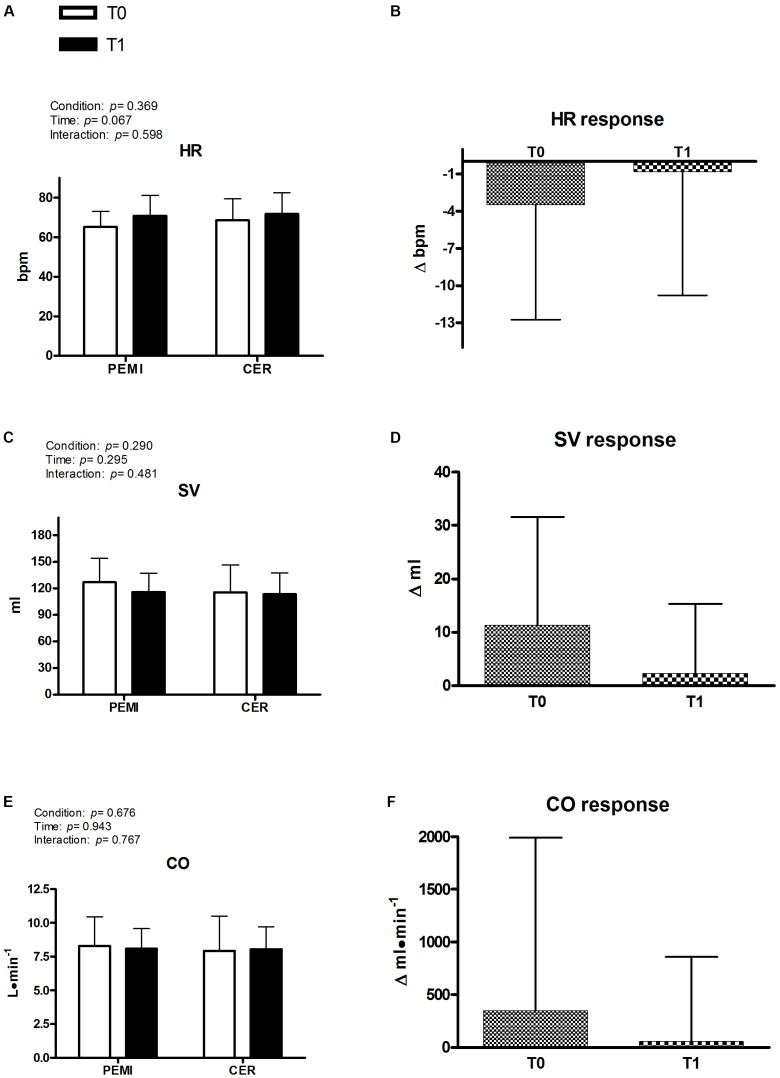
Absolute values during post-exercise muscle ischemia (PEMI) and control exercise recovery (CER) tests obtained during the third minute of recovery and response in heart rate (HR, **A,B**), stroke volume (SV, **C,D**), and cardiac output (CO, **E,F**) before (T0) and after (T1) a period of training with blood flow restriction (*n* = 17).

**FIGURE 3:**
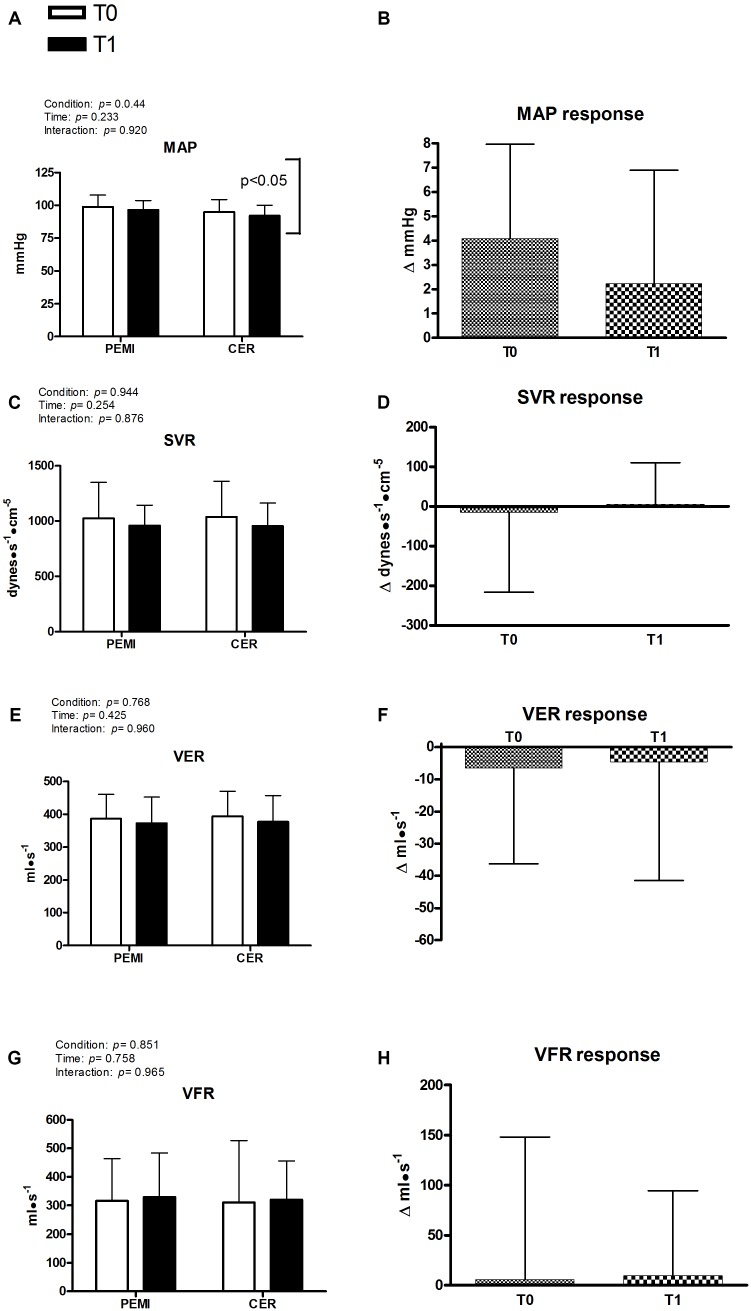
Absolute values during post-exercise muscle ischemia (PEMI) and control exercise recovery (CER) tests obtained during the third minute of recovery and response in mean arterial pressure (MAP, **A,B**), systemic vascular resistance (SVR, **C,D**), ventricular emptying rate (VER, **E,F**), and ventricular filling rate (VFR, panels **G,H**) before (T0) and after (T1) a period of training with blood flow restriction. Values are mean ± SD (*n* = 17). A vertical bracket indicates the overall main effect of condition (PEMI vs. CER). There was no interaction effect.

Figures 2, 3 exhibit hemodynamic outcomes obtained during the third minute of recovery at T0 and T1 for PEMI and CER, as well as their responses due to metaboreflex activity. Figure 2A shows that there was no difference due to condition (PEMI and CER) or time (T0 and T1) for HR, nor HR response was different between T0 and T1 (Figure 2B). Similarly, SV was not affected by condition or time (Figure 2C) and its response was similar at T0 and T1 (Figure 2D). Consequently, CO remained stable by condition and time (Figure 2E), with similar responses being observed at T1 and T0 (Figure 2F). Figure 3A shows that MAP was significantly lower during CER vs. PEMI (*P* = 0.044 for condition), without any significant time effect. Moreover, MAP (Figure 3B) was not different between T0 and T1. SVR (Figure 3C) was not influenced by time or condition, nor its response was different between T0 and T1 (Figure 3D). Similarly, VER and VFR were not affected by time and condition (Figures 3E,G) and their responses to metaboreflex were similar at T0 and T1 (Figures 3F,H).

## Discussion

This study investigated whether 1 month of BFR training was capable to reduce blood pressure during muscle metaboreflex activation in healthy subjects. Our findings indicate that training with BFR reduced MAP during dynamic handgrip (please refer to Table 2). This finding was in line with recent observations reporting hypotensive effects of BFR (Araujo et al., 2014; Maior et al., 2015; Neto et al., 2015, 2017; Sundblad et al., 2018). However, our results did not confirm the premise that MAP reduction during exercise would be due to lowered metaboreflex activity. Actually, no significant time effect was found for MAP during metaboreflex activation (Figure 3A).

Moreover, the MAP response calculated as the difference in MAP between PEMI and CER was not different between T0 and T1 (please refer to Figure 2B). Although a tendency for MAP reduction occurred when comparing T1 vs. T0 (+2.2 ± 4.6 vs. +4.0 ± 3.8), this difference was not statistically significant (*P* = 0.282). No other cardiovascular outcome was influenced by the metaboreflex activation, before or after BFR training (T1 vs. T0). These data reject the hypothesis of a blunted metaboreflex activity due to BFR training, and do not support any effect of BFR training upon muscle metaboreflex activity, at least in young and healthy male subjects.

The second objective of this experiment was to verify whether BFR training would reduce SVR and cause a shift from vasoconstriction- to flow-mediated mechanism through which the target blood pressure would be achieved during the metaboreflex activation. This initial hypothesis was also rejected, since SVR remained unaltered after training. However, it is possible to speculate that the lowered MAP during handgrip was probably due to a slight reduction in SVR at T1 vs. T0, although statistical significance was not reached (Table 2, *P* = 0.152). Further research with larger sample sizes to prevent type II error is warranted to clarify this point.

We do not have any definitive explanation for the reduced MAP during handgrip at T1 vs. T0. As previously said, this phenomenon seemed not to be mediated by the metaboreflex. It is though possible to think that the set point of baroreflex activity for the blood pressure regulation during exercise has been somehow modified by the BFR training. It has been reported that exercise training may decrease the sympathetic activity, by improving the baroreceptor control in healthy individuals, as well as in patients with cardiovascular disease (Krieger et al., 2001; La Rovere and Pinna, 2014). In the present study, it is therefore feasible to hypothesize that after BFR training the baroreflex has been more effective in buffering the exercise-related increase in sympathetic activity (Sausen et al., 2009; Delaney et al., 2010). However, we could not find previous studies investigating the effects of BFR training upon baroreflex activity. Additional research is needed to confirm this possibility.

This is probably the first study demonstrating a potential hypotensive effect of BFR training. Some previous trials have reported acute hypotension after different protocols using BFR (Araujo et al., 2014; Maior et al., 2015; Neto et al., 2015), but none addressed the chronic hypotensive effect of this modality of training. Our findings suggested that BFR training might be effective in lowering blood pressure during exercise. These data have potential clinical application in the treatment of hypertension, which should be further investigated in patients with high blood pressure.

An unexpected result was the significant reduction of resting SV at T1 in comparison with T0. We cannot provide any explanation for this outcome, since we could not find prior studies that investigated the chronic effects of BFR training upon this specific variable. However, reductions in SV have been reported during acute ischemic training (Iida et al., 2007; Downs et al., 2014; Sprick and Rickards, 2017), and some authors speculated that this effect might result from a venous pooling inhibiting venous return (Iida et al., 2007). Whatever the cause, the decrease in SV at rest seemed to be compensated by a slight increase in HR, so that CO remained unchanged.

The safety of BFR training has been questioned (Wernbom et al., 2008; Spranger et al., 2015) due to potential muscle damage, thrombosis, endothelial dysfunction, or excessive increase in blood pressure. Our results did not support the presupposition that ischemic training would be dangerous, at least when following the characteristics of the present protocol. Actually, any of these problems have been detected or reported during the intervention.

The major limitation of the present study was the relatively small sample size (*n* = 17) that completed the BRF training protocol, which prevented a better evaluation of the hemodynamic consequences of ischemic training and, in some cases, may have introduced type II error (as in the case of SVR reduction). Moreover, only men were included in our sample. We made this choice to avoid bias related to hormonal changes during the menstrual cycle, which could affect vascular responsiveness and interfere with the metaboreflex response. On the other hand, this feature limits the potential generalization of our data. The lack of a formal control group could be also considered as a methodological limitation. However, given that our main goal was to test the acute hemodynamic responses following PEMI and CER protocols, we considered that the biological test-retest evaluation would be enough – in other words, we adopted a within-individual design using the CER protocol as a control for PEMI. A formal control group composed by different subjects would add little information to the present data and might introduce inter-individual biases. Finally, it should be recognized that the fact that the individuals in the sample were physically active may have had some influence on our results. One could argue that their current exercise habits could have possibly masked the effects of BRF training upon the observed outcomes. However, considering the specificity of physiological and metabolic demands induced by successive and prolonged static contractions under BRF, it is possible to think that the general routine of physical activities of the participants was not able to modify the hemodynamic responses mediated by the metaboreflex.

## Conclusion

In conclusion, 1 month of ischemic training did not change the metaboreflex activity. MAP and SVR at rest remained unchanged after BFR training. On the other hand, the blood pressure during handgrip reduced after ischemic training, thereby suggesting a potential hypotensive effect of this training modality. Further research with larger sample sizes is warranted to better clarify the hemodynamic consequences of ischemic training and its potential application in patients with high blood pressure.

## Author Contributions

All authors contributed to data analysis and interpretation, as well as to drafting and revising the manuscript. The original study design was made by AC, PF, and RO and discussed with the other authors. AC, RO, and SR performed the data analysis.

## Conflict of Interest Statement

The authors declare that the research was conducted in the absence of any commercial or financial relationships that could be construed as a potential conflict of interest.
